# Genotyping by sequencing reveals the genetic diversity and population structure of Peruvian highland maize races

**DOI:** 10.3389/fpls.2025.1526670

**Published:** 2025-02-25

**Authors:** Carlos I. Arbizu, Isamar Bazo-Soto, Joel Flores, Rodomiro Ortiz, Raul Blas, Pedro J. García-Mendoza, Ricardo Sevilla, José Crossa, Alexander Grobman

**Affiliations:** ^1^ Centro de Investigación en Germoplasma Vegetal y Mejoramiento Genético de Plantas (CIGEMP), Universidad Nacional Toribio Rodríguez de Mendoza de Amazonas (UNTRM), Chachapoyas, Peru; ^2^ Facultad de Ingenierías y Ciencias Agrarias, Universidad Nacional Toribio Rodríguez de Mendoza de Amazonas (UNTRM), Chachapoyas, Peru; ^3^ Laboratorio de Genómica y Bioinformática, Universidad Nacional Agraria la Molina (UNALM), Lima, Peru; ^4^ Department of Plant Breeding, Swedish University of Agricultural Sciences (SLU), Alnarp, Sweden; ^5^ Facultad de Ingeniería, Universidad Nacional Autónoma de Tayacaja (UNAT), Huancavelica, Peru; ^6^ Biometrics and Statistics Unit, International Maize and Wheat Improvement Center (CIMMYT), Mexico City, Mexico

**Keywords:** germplasm, Andes, SNP markers, genetic resources, NGS

## Abstract

Peruvian maize exhibits abundant morphological diversity, with landraces cultivated from sea level (sl) up to 3,500 m above sl. Previous research based on morphological descriptors, defined at least 52 Peruvian maize races, but its genetic diversity and population structure remains largely unknown. Here, we used genotyping-by-sequencing (GBS) to obtain single nucleotide polymorphisms (SNPs) that allow inferring the genetic structure and diversity of 423 maize accessions from the genebank of Universidad Nacional Agraria la Molina (UNALM) and Universidad Nacional Autónoma de Tayacaja (UNAT). These accessions represent nine races and one sub-race, along with 15 open-pollinated lines (purple corn) and two yellow maize hybrids. It was possible to obtain 14,235 high-quality SNPs distributed along the 10 maize chromosomes of maize. Gene diversity ranged from 0.33 (sub-race Pachia) to 0.362 (race Ancashino), with race Cusco showing the lowest inbreeding coefficient (0.205) and Ancashino the highest (0.274) for the landraces. Population divergence (F_ST_) was very low (mean = 0.017), thus depicting extensive interbreeding among Peruvian maize. A cluster containing maize landraces from Ancash, Apurímac, and Ayacucho exhibited the highest genetic variability. Population structure analysis indicated that these 423 distinct genotypes can be included in 10 groups, with some maize races clustering together. Peruvian maize races failed to be recovered as monophyletic; instead, our phylogenetic tree identified two clades corresponding to the groups of the classification of the races of Peruvian maize based on their chronological origin, that is, anciently derived or primary races and lately derived or secondary races. Additionally, these two clades are also congruent with the geographic origin of these maize races, reflecting their mixed evolutionary backgrounds and constant evolution. Peruvian maize germplasm needs further investigation with modern technologies to better use them massively in breeding programs that favor agriculture mainly in the South American highlands. We also expect this work will pave a path for establishing more accurate conservation strategies for this precious crop genetic resource.

## 1 Introduction

Maize is a major global crop that is cultivated on approximately 200 million hectares and is considered a key component of food security (Erenstein et al., 2022). In several regions of the world (western South America, Mesoamerica, sub-Saharan Africa), this crop has an important social and economic value because maize is consumed daily, mainly by the poor in their single daily meal. Floury maize grows in about one million hectares in the South American Andes, where it is consumed with very little processing; that is, boiled grain (mote), fried grain without oil (cancha), or boiled ear as green corn (choclo). Migration of rural populations to urban areas dropped maize consumption by switching to more expensive products (Sevilla, 1994).

Since the publication of “The Races of Maize in Mexico” (Wellhausen et al., 1952), each country, where maize thrives, released similar books. The South American Andean races were described for Colombia (Roberts et al., 1957), Brazil and other eastern South American countries (Brieger et al., 1958), Bolivia (Ramírez et al., 1960), Perú (Grobman et al., 1961), Chile (Timothy et al., 1961), Ecuador (Timothy et al., 1963), and Venezuela (Grant et al., 1963). These investigations described 132 races in the South American Andes, that is, about 52% of the known 260 maize races (Goodman and Brown, 1988). Due to the methodology of sampling for collecting maize in the farms or rural households, maize landraces were recognized together with farmers according to their assessment for different purposes and under the name of their native language. More than half a century after these maize race books were published, we recognize the importance of the race concept to classify the diversity of this crop. Race, which is important for maize diversity classification, has been the unit of maize diversity for more than 60 years and provides means for its monitoring. For example, to the best of our knowledge, all described races remain available in Perú (Ministerio del Ambiente, 2018). To assess the current maize diversity, a new racial classification of the Peruvian maize is being conducted (Ministerio del Ambiente, 2018).

Race diversity is not an indicator of genetic structure in maize because races were described using only a few morphological characters, mostly from the ear and grain. Likewise, there are ecological and cultural criteria for classification that are not well understood. Maize farming began in Mexico about 9,000 years ago (Piperno et al., 2009). According to Kistler et al. (2018), a wild teosinte (*Zea mays* ssp. *parviglumis*) was domesticated in Mexico and thereafter spread toward the south and arrived in Peru, where it continued to expand to the Andean region and later to the Amazon region. The southwestern Amazon is, however, considered now a secondary improvement center for the partially domesticated maize (Kistler et al., 2018). Maize was grown in Peru about 6,700 years ago in the Chicama Valley, where a sample of maize with well-conserved cobs, husks, stalks, and tassels was found (Grobman et al., 2012). The macrofossil records indicated that maize included various races at that time. Grobman (1982) indicated that maize diversification began very early in human settlements. There is evidence of massive use of maize as food at Los Gavilanes, an archaeological site in Huarmey, north of Lima (Bonavia, 2008).

Andean maize possesses many beneficial (favorable) alleles to address its limitation in the production across the South American highlands (Sevilla Panizo and Holle Ostendorf, 2004; Salazar et al., 2017). The success of plant breeding depends on genetic diversity, but this must be organized, tested, and evaluated. To maintain the total maize diversity, every race of this crop should be improved. This breeding strategy ensures both variety adaptation and further adoption. Therefore, accurate classification is essential; all diversity belonging to a maize race should be understood, and all races properly defined. Precise and objective techniques and tools for these research tasks are urgently needed, as classification based on morphology, adaptation, and cultural criteria is insufficient.

The genetic structure and diversity of the races of Peruvian maize remain largely unknown. On the other hand, Vigouroux et al. (2008) used a set of almost 350 races of maize to assess their genetic and population parameters with 96 microsatellite markers, detecting four main groups: (i) highland Mexican, (ii) northern United States of America, (iii) tropical lowland, and (iv) Andean maize races. They suggested that isolation by distance could be the main factor accounting for the historical maize diversification. Further research with microsatellites suggested that the Andean group of maize displayed little mixing with other races (Bedoya et al., 2017). Genotyping-by-sequencing (GBS) is now a feasible technique for describing highly diverse and large genomes as maize (He et al., 2014). This approach is increasingly important as a cost-effective and unique tool for genomic diversity research and gene discovery in maize and provides more single nucleotide polymorphisms (SNPs) markers than SNP arrays (Wang et al., 2020).

Maize evolved rapidly due to human selection, leading to significant phenotypic changes and adaptation to various environments, such as those in Mesoamerica and the Andes. There are, however, some unanswered questions regarding maize domestication and its further evolution. The main aim of this investigation was to determine the genetic diversity and population structure of nine races and one subrace of maize grown in the highlands of Peru using SNPs covering all its 10 chromosomes generated by GBS, thus providing consistent means for understanding its classification and spread in the Peruvian Andes.

## 2 Materials and methods

### 2.1 Plant material

**Figure 1:**
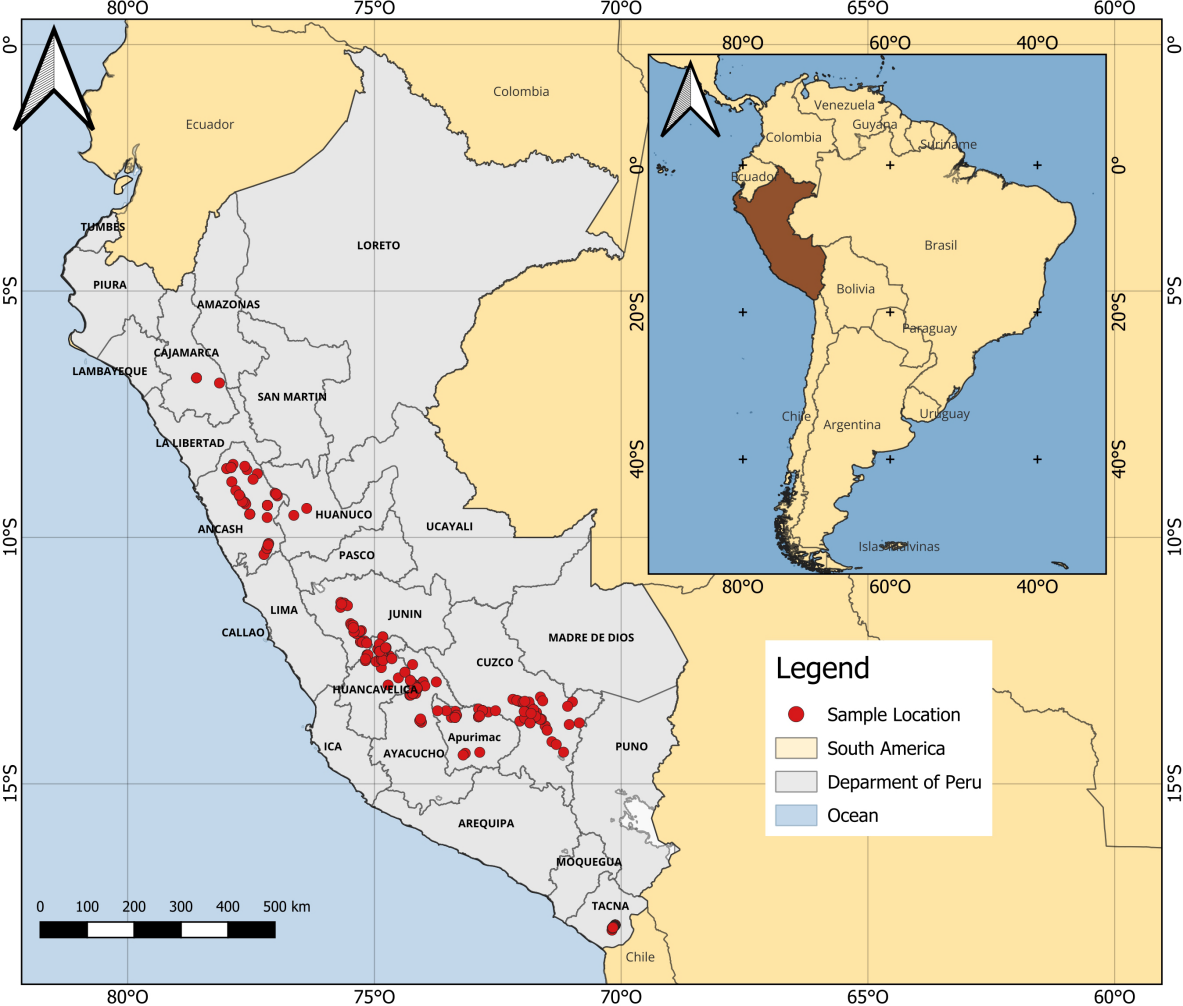
Geographic distribution of maize examined in this study. Only accessions with coordinate information available are shown.

We examined (i) 406 accessions of nine races and one sub-race of Peruvian maize that are currently cultivated in 10 Andean geographic departments of Peru, (ii) 15 open-pollinated (OP) purple maize lines, and (iii) two yellow maize hybrids (423 individuals in total) (**Figure 1**). Maize accessions were obtained from the Maize Research Program Germplasm Bank maintained at the UNALM in Lima, except for 19 accessions that were obtained from the corn research project of UNAT. One yellow dent hybrid and all purple maize were donated by Associate Prof. Hugo E. Huanuqueño (Maize Research Unit, UNALM); the other maize hybrid was a local cultivar. All possible accessions available as germplasm were employed. Further details of the maize genotypes examined in this study are available at the **Supplementary Table S1**.

### 2.2 Genotyping-by-sequencing

All 423 maize individuals (one seed per accession) were planted at UNALM, and three weeks after germination, leaf samples from one plant of each sample were collected, and total genomic DNA was extracted following Doyle and Doyle (Doyle and Doyle, 1987) protocol with some modifications. The concentration and purity of DNA samples were determined with a NanoDrop 1000 spectrophotometer. DNA samples showing absorbance ratios above 1.8 at 260/280 nm were used for further analysis. For quality determination, DNA samples were electrophoresed on 1% agarose gel, and 50 random samples were digested with *ApeKI* following the manufacturer’s protocol. Samples were sent to the University of Minnesota-Biotechnology Center for DNA sequencing.

Genotyping by sequencing libraries were developed following Elshire et al. (2011) protocol. Genomic DNA was digested with the *ApeKI* enzyme, and fragments were ligated to Illumina sequencing adapters and with sequence barcodes that are unique to each sample, which allows the recovery of sample identity for each sequenced DNA fragment after multiplexing. The pooled samples were sequenced on the Illumina NovaSeq 6000 platform, from which 100 bp single-end sequences reads were obtained. Quality of the raw data was examined with FastQC v0.11.7 software (Andrews, 2010). Thereafter, we employed the TASSEL v5.2.42 bioinformatic pipeline (Bradbury et al., 2007; Glaubitz et al., 2014) for SNPs calling with maize Zm-B73-REFERENCE-NAM-5.0 (Hufford et al., 2021) as the reference genome. Parameters employed in this pipeline were the same as in the study of Huaringa-Joaquin et al. (2023). Data curation was performed using software VCFtools v0.1.16 (Danecek et al., 2011) with the following criteria of retention: (i) minimum minor allele frequency of 0.1, (ii) number of alleles less than or equal to 2, and (iii) maximum missing data of 0.1. Additional filtering was conducted by removing SNPs in linkage disequilibrium (LD) at a threshold of *r* ^2^ = 0.2 with the function *snpgdsLDpruning* of the SNPRelate package (Zheng et al., 2012) in the R v4.2.2 (R Core Team, 2024) program. Lastly, TASSEL software was employed to convert the .vcf file to PHYLIP format with the argument *-exportType* Phylip_Inter.

### 2.3 Genetic diversity and population structure

Genetic diversity indices were calculated for each race of maize and for the OP and hybrid cultivars using the *adegenet* v2.1.10 (Jombart, 2008; Jombart and Ahmed, 2011) and the HIERFSTAT v0.5-11 (Goudet, 2005) R packages. Bootstrapped 95% confidence intervals were estimated by running 10,000 bootstraps (BS) using HIERFSTAT package to determine if F_IS_ was significantly different from zero. A maximum likelihood (ML) tree was constructed using the .phy file with the multi-threaded version of the program RAxML v8.2.11 (Stamatakis, 2014), *raxmlHPC-PTHREADS*, with the rapid bootstrapping algorithm and a total of 100 nonparametric BS inferences. Model *ASC_GTRGAMMA* with the ascertainment correction of Lewis (Lewis, 2001) was also considered, and the resulting tree was plotted with the ggtree (Yu et al., 2017) R package. Moreover, genetic distances based on Provesti´s coefficient (Prevosti et al., 1975) were calculated, and then a dendrogram was generated using the neighbor-joining clustering algorithm with 100 BS replicates from the *poppr* v.1.1.4 (Kamvar et al., 2014, 2015) package in R. A principal coordinate analysis (PCoA) was performed with the *dudi.pco* function of the ade4 v1.7.22 (Dray and Dufour, 2007) package in R. To determine the population structure, first the filtered .vcf file was converted into .str format with VCFtools and PGDSpider v2.1.1.5 (Lischer and Excoffier, 2012) programs.

We then employed the Bayesian clustering program STRUCTURE v2.3.4 (Pritchard et al., 2000) with populations (K) of 1 to 25 and 10 replicates. A burn-in length of 50,000 with 100,000 Monte Carlo iterations was considered, and the optimal K value was estimated by the Evanno method (Evanno et al., 2005). Population structure was visualized with POPHELPER v2.3.1 (Francis, 2017) R package. Genetic diversity indices were also estimated for the clusters determined by STRUCTURE. The Evanno method determined that the best K (number of populations) is two for our data set, and the next two largest peaks are at *K* = 4 and *K* = 10 (**Supplementary Figure S1**). Previous research in capirona (Saldaña et al., 2021), carrot (Iorizzo et al., 2013; Arbizu et al., 2016), and maize (Vigouroux et al., 2008) found a false highest peak at *K* = 2 in population structure analysis as the null hypothesis of no structure (*K* = 1) was strongly rejected. In addition, Waples and Gaggiotti (2006); Frantz et al. (2009), and Janes et al. (2017) indicated that the Evanno method tends to underestimate the number of genetic clusters. Hence, it is very likely the second highest peak obtained with our dataset of 14,235 SNPs (*K* = 4) is caused by a strong rejection of the hypothesis of three clusters only. Furthermore, the ML value was obtained at *K* = 10 (**Supplementary Figure S2**), which is concordant also with our ML analyses of the Peruvian maize. Hence, we decided to discuss our results with *K* = 10.

An analysis of molecular variance (AMOVA) with the *poppr* (Kamvar et al., 2014) package in R to determine the sources of genetic variance within and among the races of maize was also conducted. To evaluate statistical significance, a randomization test with 999 permutations was performed using the *randtest* function of the ade4 (Dray and Dufour, 2007) package. Finally, a pairwise fixation index (F_ST_) was estimated using the R package HIERFSTAT, according to Weir and Cockerham (1984).

## 3 Results

### 3.1 Sequencing analysis and SNPs distribution

After filtering out the raw reads, the total demultiplexed reads for all 423 genotypes were 1,566.7 M with good barcoded reads representing 99.9%, and the average read per accession was 3.7 M. A total of 5,010,502 tags were identified, of which 86.4% uniquely aligned to the maize reference genome. Next, we detected a total of 1,002,078 raw SNPs after using the TASSEL software, and we kept 31,132 SNPs after filtering with VCFtools program. A set of 14,235 SNPs distributed across the 10 chromosomes of maize was selected after LD pruning in R, which was used for subsequent genetic structure and diversity analysis. The highest and lowest numbers of physically mapped SNPs were identified in chromosome 1 (2170, 15.2%) and 10 (954, 6.7%), respectively (**Table 1**). The 10 maize chromosomes exhibited a very consistent distribution of SNP markers that spanned virtually the whole genome, showing a low SNP density near the centromeres, whereas the telomere region exhibited a high density of SNPs (**Supplementary Figure S3**). Chromosome 1 possessed the highest density (7.04 SNPs/Mb), and chromosome 10, with 6.26 SNPs/Mb, the lowest (**Supplementary Figure S3**).

**Table 1:** Genome-wide distribution and density of 14,235 single nucleotide polymorphisms (SNPs) across the 10 chromosomes of maize.

Chromosome	SNP #	SNP %	Total length (Mb)	Density (SNPs/Mb)
1	2170	15.2	308.45	7.04
2	1793	12.6	243.68	7.36
3	1631	11.5	238.02	6.85
5	1615	11.3	226.35	7.13
4	1410	9.9	250.33	5.63
7	1260	8.9	185.81	6.78
8	1229	8.6	182.41	6.74
6	1109	7.8	181.36	6.12
9	1064	7.5	163	6.53
10	954	6.7	152.44	6.26

### 3.2 Population structure

**Figure 2:**
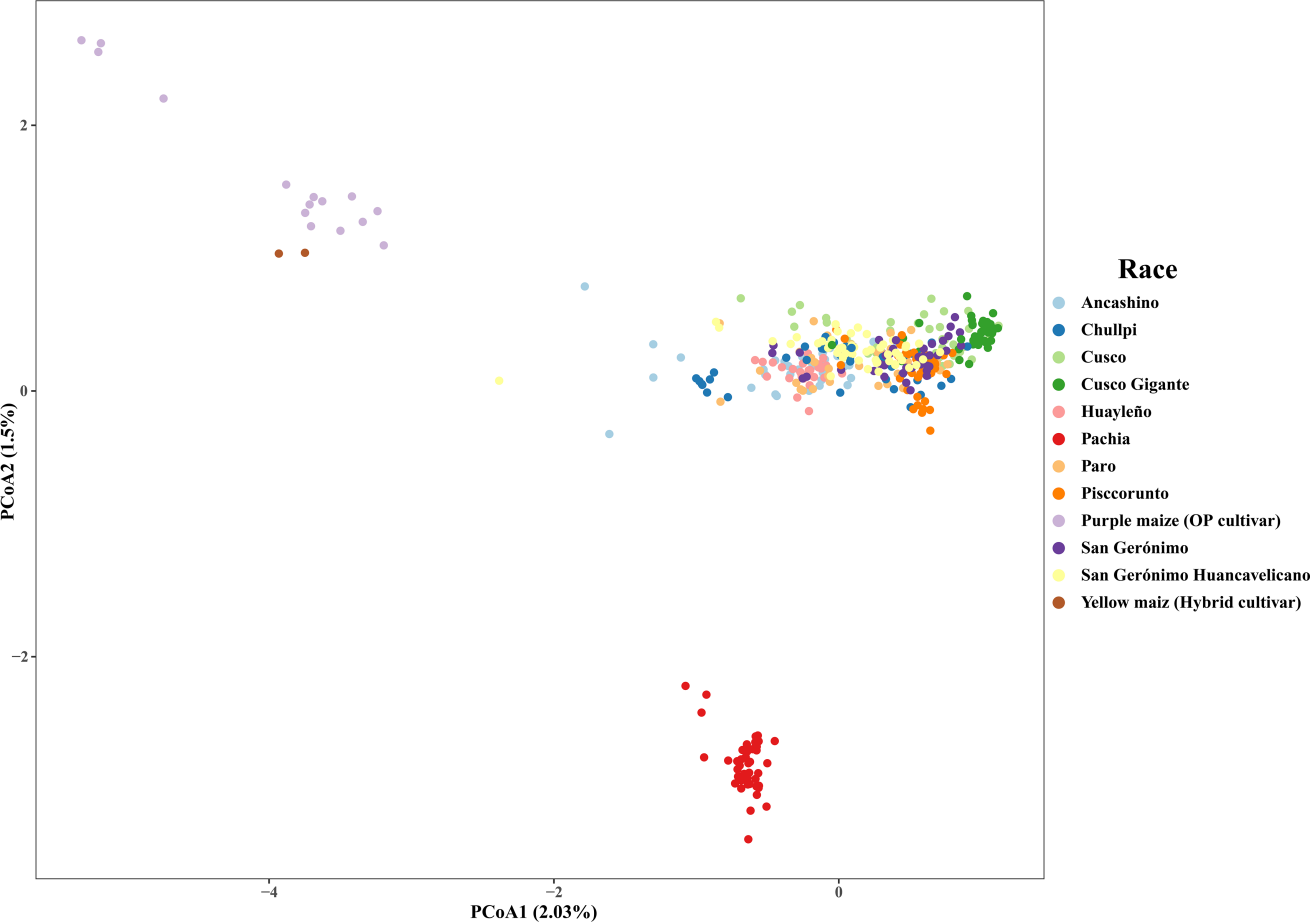
Principal coordinate analysis of 423 accessions of Peruvian maize germplasm using 14,235 single nucleotide polymorphisms (SNPs). Percentages on the axis represent the variance explained by each coordinate.

The PCoA showed that the first and second axis explained 2.0% and 1.5% of the variance, respectively. Sub-race Pachia and the two types of improved maize (purple and yellow cultivars) are separated into two well-distinctive groups. On the contrary, there is not a consistent grouping of the other amylaceous maize. Accessions of race Cusco Gigante are closely related to some individuals of Cusco, and races Ancashino, Chullpi, Huayleño, and Paro are grouping together, but without a clear resolution (**Figure 2**). Deletion of improved maize slightly changed the structure in the PCoA, showing that most accessions of race Chullpi are separated, and race Cusco Gigante and most accessions of Cusco are roughly grouping together (**Supplementary Figure S4**). When accessions of sub-race Pachia were also deleted, it was possible to detect a better grouping of race Cusco Gigante. Similarly, races Ancashino, Huayleño, and Paro tend to cluster together. In addition, eight accessions of race Chullpi are separated from the other individuals of maize; some accessions of race San Gerónimo Huancavelicano are separated too but mixed with very few individuals of Pisccorunto and Cusco Gigante (**Supplementary Figure S5**).

**Figure 3:**
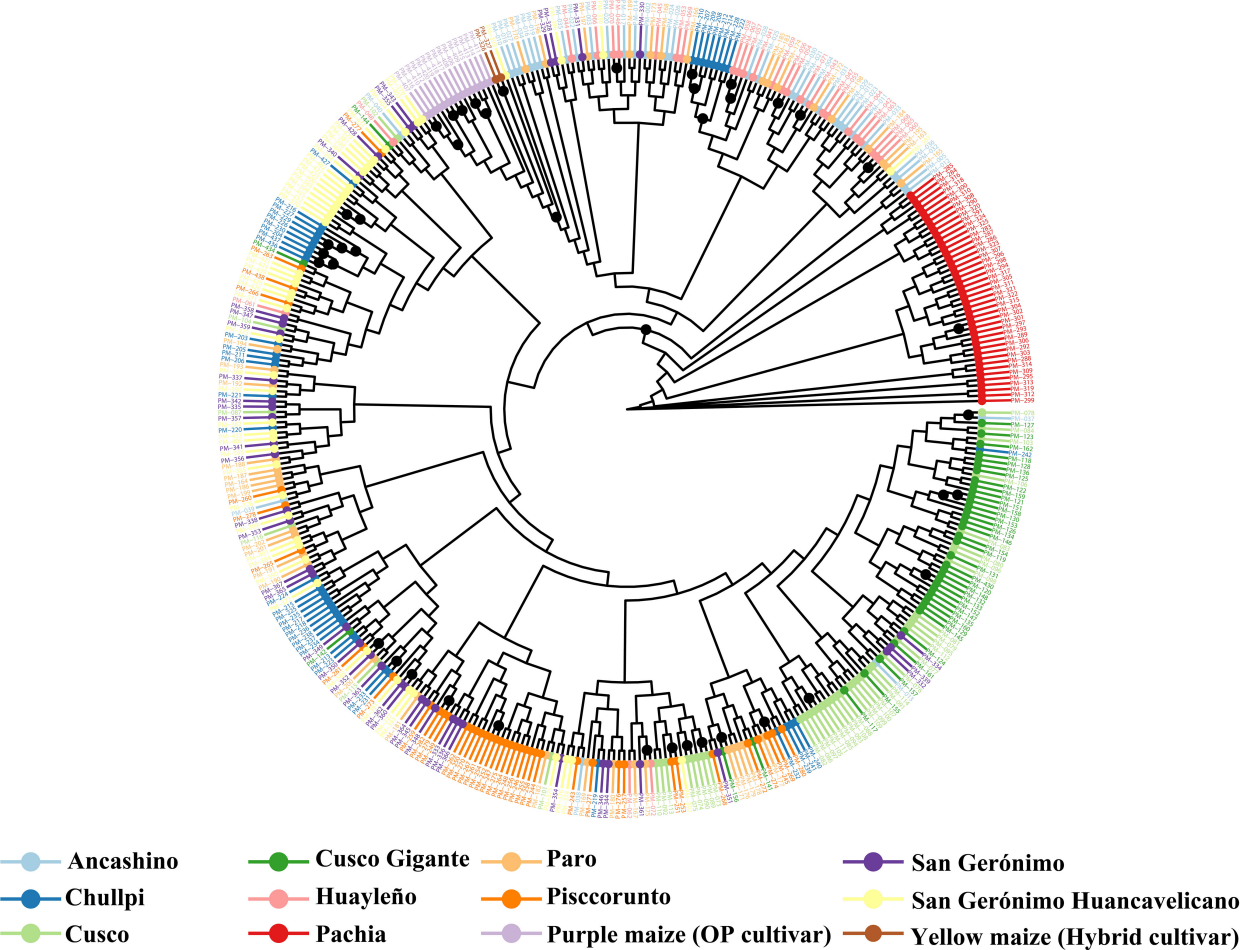
Maximum likelihood reconstruction of 423 accessions of Peruvian maize germplasm using 14,235 single nucleotide polymorphisms (SNPs). Round symbol on nodes represents bootstrap support, with only values higher than 90% shown.

Accessions of race Ancashino and Huayleño are grouping together, but some other accessions are intermixed in this group. The same feature was shown by another group of some accessions of race Pisccorunto. However, most accessions cannot be clearly separated by race criteria. Our ML tree recovered two main clades containing the following maize landraces: (CR1) almost all accessions of Ancashino, Huayleño, and Paro, some accessions of Chullpi, and a few of San Gerónimo and San Gerónimo Huancavelicano, and (CR2) all accessions of Cusco Gigante, Cusco, and Pisccorunto, some accessions of Chullpi, San Gerónimo, and San Gerónimo Huancavelicano (**Figure 3**).

In addition, all individuals of improved maize were placed in a subcluster within CR1. Interestingly, the CR1 mainly comprises the anciently derived or primary races (ADPR) of maize in Peru, and CR2 consists of the lately derived or secondary races (LDSR), according to the classification described by Grobman et al. (1961) (**Figure 3**). There are, however, few exceptions: (i) races Cusco and Pisccorunto are considered an ADPR, but they are not within the CR1 clade but in CR2 clade, (ii) initially defined as an imperfectly defined race by Grobman et al. (1961), San Gerónimo should be considered as an LDSR. A clade of two accessions of Ancashino (PM-005, PM-012) and one of Paro (PM-165) races is sister to those two clades, and sister to them there is a grade comprising sub-race Pachia. Our ML tree also resolved a subclade within CR2 containing almost all accessions of race Cusco Gigante and Cusco. Moreover, we observed three subclusters of race Chullpi, one of them with above 90% BS within CR1. One grade comprising 10 accessions of race San Gerónimo Huancavelicano, including one Chullpi maize (PM-427), was also detected. For most accessions labeled by their race, a consistent grouping pattern was not noticed. The topology of our neighbor-joining dendrogram differs to some degree to the ML tree. Only clade CR1 was recovered, and a polytomy was present (**Supplementary Figure S6**).

A clear grouping was not observed when maize accessions were labeled based on their Peruvian geographic department of origin, except for accessions from Tacna, who form a grade with above 90% BS. Maize from Ancash also showed another grade, but accessions from other locations are also intermingled; similarly, another grade formed by maize from Huancavelica and Junín (**Supplementary Figure S7**). Interestingly, when accessions were labeled according to their geographic zone of origin (north, center, south) in Peru, our ML tree revealed the following two clades: (CZ1) individuals from the northern Andes (Ancash and Cajamarca) and purple maize OP lines obtained in Lima, (CZ2) individuals from the center (Huancavelica, Huánuco, Junín) and southern (Apurímac, Ayacucho, Cusco, Moquegua) of the Peruvian Andes; both also contained within their corresponding subclades. These two clades possess a BS <90%, and very few accessions from other geographic zones are intermingled. A similar pattern was detected in our PCoA (**Supplementary Figure S8**).

**Figure 4:**
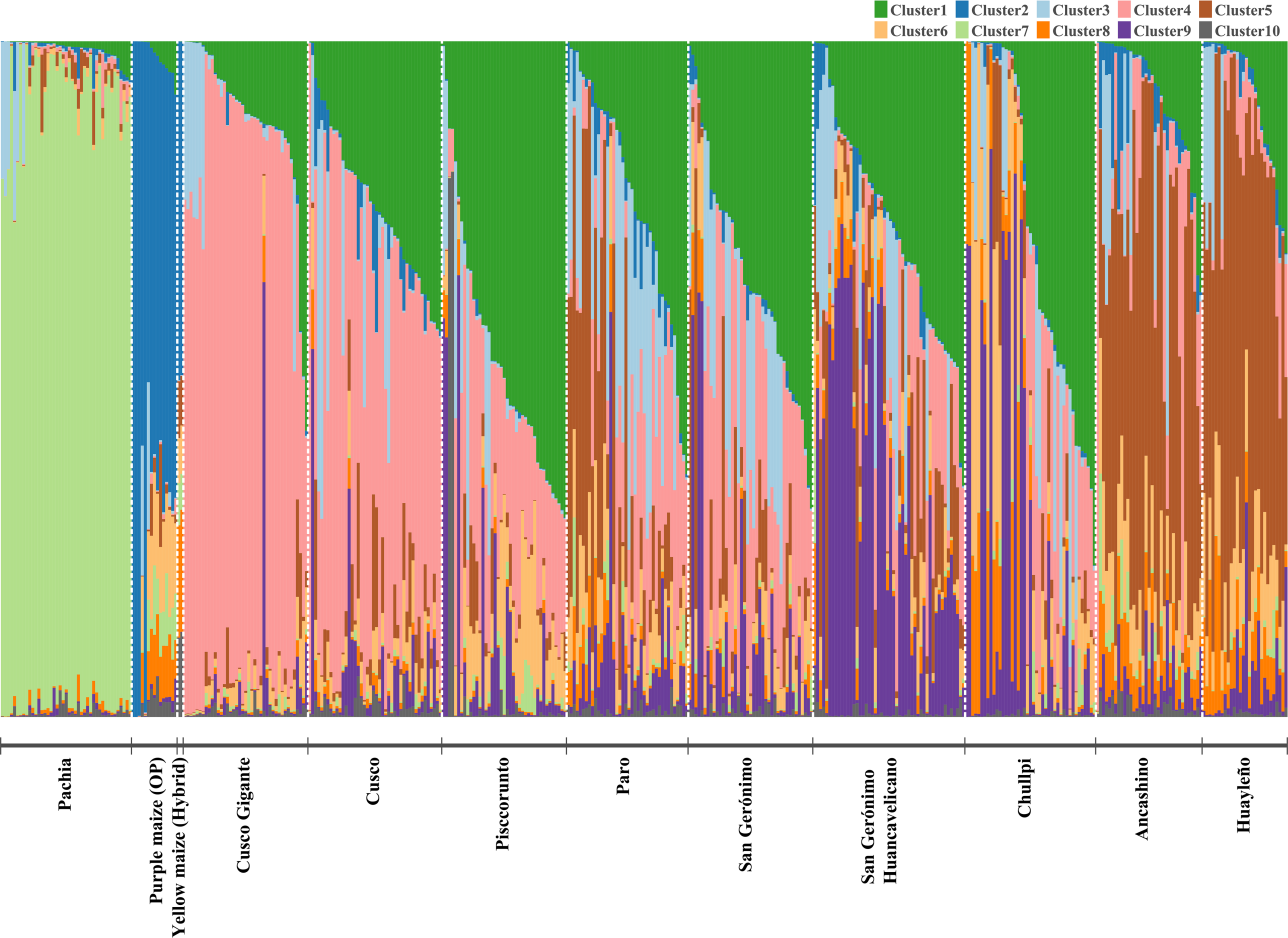
Population structure of 423 maize accessions based on 14,235 single nucleotide polymorphisms (SNPs). Each accession is represented by a vertical bar, and each color corresponds to a population (10 in total).

STRUCTURE analysis showed abundant admixture, except for the accession of sub-race Pachia, whose accessions were placed in cluster (C) 7, thus exhibiting very low admixture (**Figure 4**). Furthermore, Cusco Gigante and Cusco races are clustering together; that is, most of those accessions are within cluster 4. Like Ancashino and Huayleño, which are forming a group (cluster 5) but with some degree of admixture, improved maize is also clustering together (C2). Chullpi accessions are mainly distributed between clusters 1 and 9, and race Paro was placed in clusters 1, 3, and 5. San Gerónimo Huancavelicano is grouped within clusters 1 and 9 mainly, whereas race San Gerónimo was placed mainly in cluster 1. For *K* = 2, yellow (hybrid) and purple maize (OP) were the only groups (C2) clearly differentiated from the other maize. Admixture was also observed for *K* = 4, and similar to *K* = 2 and *K* = 10, bred maize clearly grouped together (C3). Furthermore, subrace Pachia was also differentiated (C1), and a grouping of most accessions of races Cusco Gigante, Cusco, Pisccorunto, Paro, and San Gerónimo was detected (C4). On the other hand, C2 consisted of most accessions of races Ancashino, Huayleño, Chullpi, and San Gerónimo Huancavelicano (**Supplementary Figure S9**). An erratic behavior for ML values for K exceeding 11 was noticed (**Supplementary Figure S2**). This requires further methodological research, as perhaps large SNP data sets require more iterations in burn-in.

There was not a clear cluster assignation when accessions were labeled according to their geographic origin, except for maize from Ancash, Huánuco, Moquegua, Tacna, and Lima (bred maize). The geographic zone criterion of clustering exhibited that most accessions from northern Peru are grouped together (C5), while those from Lima were placed in cluster 2. Maize accessions from the center of Peru were mainly grouped within clusters 1, 4, and 9. Clusters 1, 4, and 7 contained accessions of maize from southern Perú (**Supplementary Table S2**, **Supplementary Figure S10**). Clusters 8 and 10 possessed one and two individuals only, respectively; therefore, results of these clusters will not be described.

Fixation indices (F_ST_) were very low in general. Population divergence between yellow maize (improved maize) and Cusco Gigante revealed the highest genetic difference (0.18), while races Huayleño and Ancashino exhibited the lowest (0.001), which might not be significantly different from zero (**Supplementary Table S3**). Furthermore, the greatest genetic variation was observed within races of Peruvian maize (95.48%), while 4.52% was reported for between races, according to our AMOVA. A significant PhiPT value of 0.045 (*p*-value < 0.001) was obtained (**Table 2**).

**Table 2:** Analysis of molecular variance of the genetic variation for 423 accessions of Peruvian maize germplasm using 14,235 SNPs.

Source of variation	*df*	SS	MS	Est. var.	%	PhiPT	*p*-value
Between races	11	179,018.3	16,274.39	290.99	4.52	0.045	0.001
Within races	411	2,526,998.7	6148.42	6148.42	95.48		
Total	422	2,706,017.0	6412.36	6439.41	100		

*df*, degree of freedom; SS, sum of squares; MS, mean squares; Est. Var., estimated variance; %, percentage of genetic variation; PhiPT, differentiation statistics; *p*-value, probability of accepting the null hypothesis.

### 3.3 Genetic diversity

The nine races and one subrace of Peruvian maize examined in this work showed a very similar number of different alleles; the allelic richness ranged from 1.33 ± 0.15 (subrace Pachia) to 1.36 ± 0.12 (race Ancashino). In addition, the sub-race Pachia possessed the lowest observed heterozygosity (H_O_) (0.25 ± 0.17), whereas the race Cusco had the highest (0.28 ± 0.16), and the expected heterozygosity (H_E_, genetic diversity) ranged from 0.33 ± 0.14 (Pachia) to 0.36 ± 0.12 (Ancashino). On the other hand, Cusco (0.21) exhibited the lowest inbreeding coefficient (F_IS_), while Ancashino the highest (0.27) (**Table 2**). Cluster 2 (1.33 ± 0.16) and 5 (1.36 ± 0.11) possessed the lowest and highest allelic richness, respectively. The lowest H_O_ was exhibited by C2 (0.2 ± 0.16), whereas the highest was by C1 (0.28 ± 0.15). In addition, C2 (0.33 ± 0.16) also showed the lowest H_E_, while C5 (0.36 ± 0.11) showed the highest. Interestingly, clusters containing mainly maize landraces from Apurímac (C3) and Ayacucho (C1) possessed very high levels of genetic diversity (0.35–0.36). F_IS_ ranged from 0.21 (C4) to 0.4 (C2) (**Table 3**).

**Table 3:** Genetic diversity indices of Peruvian maize germplasm based on 14,235 single nucleotide polymorphisms (SNPs).

Race (or cultivar type)	Number of accessions	N_A_	A_R_	H_O_	H_E_	F_IS_
Ancashino	35	2.000 ± 0.01	1.360 ± 0.12	0.263 ± 0.16	0.362 ± 0.12	0.274 [0.27;0.28]
Chullpi	43	1.999 ± 0.02	1.353 ± 0.12	0.264 ± 0.16	0.354 ± 0.12	0.254 [0.25;0.26]
Cusco	44	2.000 ± 0.02	1.349 ± 0.12	0.278 ± 0.16	0.350 ± 0.12	0.205 [0.2;0.21]
Cusco Gigante	41	1.996 ± 0.06	1.336 ± 0.13	0.262 ± 0.17	0.337 ± 0.13	0.223 [0.22;0.23]
Huayleño	28	1.998 ± 0.05	1.355 ± 0.13	0.265 ± 0.16	0.357 ± 0.13	0.257 [0.25;0.26]
Pachia	43	1.988 ± 0.11	1.332 ± 0.15	0.249 ± 0.17	0.333 ± 0.14	0.253 [0.25;0.26]
Paro	40	2.000 ± 0.01	1.356 ± 0.12	0.277 ± 0.16	0.357 ± 0.12	0.225 [0.22;0.23]
Pisccorunto	41	1.999 ± 0.03	1.346 ± 0.12	0.274 ± 0.17	0.347 ± 0.12	0.211 [0.2;0.22]
Purple corn (OP)	15	1.914 ± 0.28	1.313 ± 0.17	0.190 ± 0.17	0.318 ± 0.17	0.401 [0.39;0.41]
San Gerónimo	41	1.999 ± 0.02	1.350 ± 0.12	0.267 ± 0.16	0.351 ± 0.12	0.238 [0.23;0.24]
San Gerónimo Huancavelicano	50	2.000 ± 0.01	1.354 ± 0.12	0.270 ± 0.16	0.355 ± 0.12	0.239 [0.23;0.24]
Yellow maize (hybrid)	2	1.440 ± 0.52	NA	0.230	0.297	0.227
Cluster (C)						
C1	112	2.000 ± 0	1.349 ± 0.12	0.274 ± 0.15	0.350 ± 0.12	0.217 [0.21;0.22]
C2	18	1.948 ± 0.22	1.325 ± 0.16	0.198 ± 0.16	0.329 ± 0.16	0.399 [0.39;0.4]
C3	31	1.999 ± 0.03	1.353 ± 0.12	0.263 ± 0.17	0.355 ± 0.12	0.258 [0.25;0.26]
C4	91	2.000 ± 0.01	1.342 ± 0.12	0.272 ± 0.16	0.343 ± 0.12	0.206 [0.2;0.21]
C5	76	2.000 ± 0	1.359 ± 0.11	0.268 ± 0.15	0.360 ± 0.11	0.255 [0.25;0.26]
C6	7	1.852 ± 0.35	1.326 ± 0.18	0.247 ± 0.22	0.333 ± 0.19	0.258 [0.25;0.27]
C7	43	1.988 ± 0.11	1.332 ± 0.14	0.249 ± 0.17	0.333 ± 0.14	0.253 [0.25;0.26]
C8	1	0.019 ± 0.14	NA	0.004 ± 0.06	0.004	-0.001
C9	42	1.998 ± 0.04	1.346 ± 0.13	0.262 ± 0.16	0.348 ± 0.13	0.246 [0.24;0.25]
C10	2	1.342 ± 0.49	NA	0.250 ± 0.38	0.190 ± 0.26	-0.311 [-0.43;-0.39]

N_A_, number of different alleles; A_R_, allelic richness; H_O_, observed heterozygosity; H_E_, expected heterozygosity; F_IS_, inbreeding coefficient. Values after ± represent standard deviation. Number in brackets correspond to 95% confidence intervals. NA, not applicable as number of samples is limited.

## 4 Discussion

The foundation for crop improvement lies in genetic diversity (Hoisington et al., 1999; Bhandari et al., 2017), which can be assessed by DNA (molecular) markers like SNPs. Analyzing the molecular genetic variation in germplasm provides valuable insights into allelic richness, population structure, and diversity parameters. This information helps plant breeders utilize genetic resources more effectively, reducing the need for extensive pre-breeding tasks when developing new cultivars (Govindaraj et al., 2015). In recent years, due to the advances in next-generation sequencing (NGS), GBS has emerged as a promising genomic approach for estimating plant genetic diversity and population structure on a genome-wide scale and has been successfully employed, *inter alia*, in *Brassica* (McAlvay et al., 2021), *Daucus* (Arbizu et al., 2016; Mezghani et al., 2018; Martínez-Flores et al., 2020), finger millet (Brhane et al., 2022), maize (Swarts et al., 2017; Shu et al., 2021; Dube et al., 2023), spruce (Korecký et al., 2021), wheat (Alipour et al., 2017), and watermelon (Lee et al., 2019). However, GBS has not been used so far for genotyping Peruvian races of maize, whose morphological diversity seems to be the largest worldwide (Grobman et al., 1961; Ministerio del Ambiente, 2018). Studies on Peruvian maize have primarily focused on morpho-agronomic characteristics (Ortiz et al., 2008b, 2008c; López Alejandría, 2011; Chavarry Gómez, 2014; Macuri Núñez, 2016; Gamarra Sánchez et al., 2020; Chambergo Zúñiga, 2021; Garcia Mendoza et al., 2021; Mamani-Huarcaya et al., 2022; Prieto Rosales and Manayay Sánchez, 2022; García-Mendoza et al., 2023), leaving their molecular composition largely unexplored. Herein, we determined the gene diversity and composition of Peruvian maize races from the Andean highlands by means of SNP markers spanning each chromosome.

Unfortunately, despite the significant diversity within Peruvian maize germplasm, knowledge of its genetic components remains very limited. Catalán et al. (2019) reported a high level of variability using eight microsatellites in 83 accessions of six races of maize from Cusco. The genetic diversity of the nine races and one subrace of maize assessed in this study is very high, which is concordant with its improvement status (i.e., landraces), as reported for other landraces of beans (Özkan et al., 2022), peas (Martin-Sanz et al., 2011), squash (Lorello et al., 2020), tarwi (Huaringa-Joaquin et al., 2023), or wheat (Tehseen et al., 2022), among others. Our genetic diversity indices align with other studies on maize landraces. For example, Warburton et al. (2008) examined the genetic diversity of 24 maize landraces from Mexico with 25 simple sequence repeats (SSR) and reported a total gene diversity of 0.61 across all populations. Similarly, Herrera-Saucedo et al. (2019) determined the genetic variability of 63 native maize accessions from northern Mexico using 31 SSRs, reporting an expected heterozygosity of 0.68. A study of 30 maize landrace accessions from the southern Andean region of South America using 22 SSRs showed a genetic diversity of 0.72 (Rivas et al., 2022).

In a more comprehensive study (Vigouroux et al., 2008), employing 96 SSRs that encompass most of the described races in the American continent, 136 accessions of 47 races of Peruvian maize were included, and these, together with other maize from Ecuador and Bolivia (a total of 235 plants), possessed a total gene diversity of 0.71. Here, we determined that the genetic diversity of the Peruvian maize (0.35) from more diverse geographic regions is higher than the value reported for 46 Mexican landraces (161 accessions) of maize using SNPs (0.311) (Arteaga et al., 2016), demonstrating that Peru possesses one of the largest genetic diversities of amylaceous maize, pointing to the fact that the central Andean region possesses abundant maize genetic variability. A recent study (Arca et al., 2023) found that gene diversity of landraces from seven countries from South America, assessed with 23,412 SNPs, was slightly lower (0.323 ± 0.007) than landraces from Central America and Mexico (0.328 ± 0.006). The higher gene diversity reported with SSR compared to SNP markers may be due to the multi-allelic nature and higher level of polymorphism of SSR compared to bi-allelic SNP. However, SNPs are more reliable for inferring genome-wide genetic diversity, as demonstrated by previous work (Fischer et al., 2017; García et al., 2018). Cluster 5, containing mainly landraces of Ancashino, Hualyeño, and Paro from Ancash, Apurímac, and Ayacucho, exhibited the highest gene diversity. In addition, C3 and C1, which are mainly comprised of maize landraces from Apurímac and Ayacucho, respectively, also showed very high levels of genetic variability. This result is in agreement with Salhuana (2004), as he indicated that maize from the geographical departments of Ancash and Ayacucho exhibited a greater degree of variability and a more contrasting pattern of allele frequencies than all other maize from other Peruvian locations. It is very likely maize landraces from Ancash, Apurímac, and Ayacucho were involved in the origin of Andean maize. Furthermore, Ancash is the worldwide center of brown and red coloration of pericarp and cob of maize (Grobman et al., 1961; Salhuana, 2004).

Consistent with previous investigations (Warburton et al., 2008; Salazar et al., 2017), bred maize is clearly separated from Peruvian maize landraces, which is explained by their intensity of selection. The well-defined grouping of accessions of sub-race Pachia may be explained in the light of its cultivation in a restricted area in southern Peru (Valley of Pachia, Tacna). Although Grobman et al. (1961) indicated that this sub-race derived from the race Arequipeño, which is a lately derived race, our molecular data, however, do not support this fact, as Pachia was not placed within the CR2 clade. Instead, it is more likely related to race Coruca, which also grows in Tacna and is similar to a floury maize landrace Choclero from Chile (Grobman et al., 1961). Further research is needed, including maize samples from other southern Peruvian regions (Arequipa, Moquegua, Puno, and Tacna), as landraces of maize cultivated in Tacna show tolerance to high levels of boron (Mamani-Huarcaya et al., 2020, 2022), which is a trait of interest for breeding maize for locations with high levels of this element in soil and irrigation water. Landrace Cabanita, widely grown in Arequipa, also shows potential as a source of phenolic compounds with *in-vitro* antioxidant capacity (Fuentes-Cardenas et al., 2022). Races Cusco Gigante and Cusco tend to group together as they are mainly cultivated in Cusco. Additionally, races Ancashino and Huayleño, both sympatrically distributed in northern Peru (Ancash), group together and possess a very low F_ST_, suggesting they evolved simultaneously. Hybridization likely plays a role in the grouping of these races, as noted by Grobman et al. (1961).

Even though the other Peruvian maize races are morphologically distinct, our GBS dataset failed to support them as monophyletic, which agrees with other research that evaluated Peruvian germplasm (Matsuoka et al., 2002; Vigouroux et al., 2008; Bracco et al., 2016; Bedoya et al., 2017; Arca et al., 2023). Similarly, Mexican maize races do not form distinct clusters (Arteaga et al., 2016). However, Caldu-Primo et al. (2017) were able to distinguish Mexican maize races based on a high F_ST_ SNP dataset. Population structure analysis clustered maize races from the American continent based on geographic origin, with the Peruvian germplasm contained within a clade named “Andean” (Matsuoka et al., 2002; Vigouroux et al., 2008; Bracco et al., 2016; Bedoya et al., 2017; Arca et al., 2023). More consistent clustering was observed when Peruvian races of maize were labeled according to their geographical zones of origin, identifying CZ1 and CZ2. This mixing among maize landraces is likely due to extensive gene flow within these zones explained by their proximities and frequent seed exchange, which is a common practice in the Peruvian Andes. However, Peruvian maize farmers in the Andes usually dynamize seed flow between families and rural communities and conduct selection within their populations to maintain the morphological characteristics of their landraces. Our ML tree mostly agrees with the classification of Peruvian maize races based on the chronological origin described by Grobman et al. (1961) as it was possible to reconstruct the ADPR (CR1) and LDSR (CR2) clades. Races Chullpi and Paro possess very low F_ST_ (0.004), reflecting very low genetic differentiation. Moreover, the phylogenetic position of race Chullpi warrants additional research to determine its origin. The similar traits that race Chullpi from different geographical departments of Peru exhibits are very likely due to environmental pressures, as the climatic gradients of the Andes are laboratories of constant plant evolution. Similarly, further research is needed for races Cusco Gigante, Cusco, Pisccorunto, San Gerónimo, and San Gerónimo Huancavelicano, as it is very likely their phenotype is a result of evolution in the Andes of Peru of a set of novel or derived traits.

A more detailed morphological evaluation is needed for the races of Peruvian maize to identify morphotypes and determine their phenetic plasticity. It is very likely that two accessions of Ancashino (PM-005, PM-012) and one of Paro (PM-165) contributed to the origin of other maize races evaluated in this work, as these individuals form a sister clade to CR1 and CR2. Both races are considered ADPR, directly derived from the primitive races (PR), as described by Grobman et al. (1961). Therefore, it is very likely these three accessions may still possess genetic signatures of PR. However, further research is necessary, including samples from a wider geographical area, for more conclusive results. The position of purple maize OP cultivars within CR1 is explained by its origin in race Kculli, classified as ADPR by Grobman et al. (1961). The close relation among purple (OP) and yellow maize (hybrid) is due to the use of the latter at UNALM to enhance the yield performance of OP lines.

The vast diversity exhibited by maize races in Peru is crucial for research in plant genetic resources (Ortiz et al., 2008a). However, the lack of relevant information on the genetic diversity of conserved plant material hinders the use of accessions preserved in germplasm banks (Ortiz and Engels, 2018; McCouch et al., 2020). To address this gap for Peruvian maize, we suggest that genomic tools can facilitate the characterization and utilization of this invaluable plant genetic resource, as emphasized by Mascher et al. (2019). This approach is particularly important considering that amylaceous maize in Peru are landraces, dynamic populations in constant evolution in the Andes. Moreover, Peruvian maize germplasm requires special attention as a ~5500 cal. BP maize cob from northern Peru (Grobman et al., 2012) was the only sample without the *Zea mays* ssp. *mexicana* ancestry in a recent study (Yang et al., 2023), shedding light on the origin of maize in the Central Andean region. Swarts et al. (2017) did find introgression with *Z*. *mays* spp. *mexicana* in modern South American landraces. On the other hand, we expect our study will provide useful guides to researchers and decision makers for establishing a strong conservation strategy and dynamic utilization soon for Peruvian maize germplasm.

## Data Availability

The dataset generated and/or analyzed during the current study are available in the Dryad repository, https://datadryad.org/stash/share/4OI0AXLoEIDsbSSLvoOdXrhzSyMUAow9x0elGjhyPmQ. Additional information is presented in the Supplementary Data file accompanying this article.

## Supplementary material

Supplementary Figure 1 Plot of Delta K (ΔK). Twenty-five populations were considered in a data set of 14,235 SNPs and 423 accessions of Peruvian maize germplasm.

Supplementary Figure 2 Plot of the log likelihood for K ranging from 1 to 25.

Supplementary Figure 3 Density and distribution of 14,235 single nucleotide polymorphism markers on the 10 maize chromosomes. Red bars denote the end of chromosome; nt refers to nucleotide.

Supplementary Figure 4 Principal coordinate analysis of 406 accessions of Peruvian maize germplasm (improved maize not included) using 14,235 single nucleotide polymorphism markers. Percentages on the axis represent the variance explained by each coordinate.

Supplementary Figure 5 Principal coordinate analysis of 363 accessions of Peruvian maize germplasm (improved maize and sub-race Pachia not included) using 14,235 single nucleotide polymorphism markers. Percentages on the axis represent the variance explained by each coordinate.

Supplementary Figure 6 Dendrogram based on Provesti’s genetic distance and the neighbor-joining clustering method of 423 accessions of Peruvian maize germplasm using 14,235 single nucleotide polymorphisms markers. Numbers above the branches represent bootstrap values, with only values higher than 90% shown.

Supplementary Figure 7
**(A)** Principal coordinate analysis of 423 accessions of Peruvian maize germplasm using 14,235 single nucleotide polymorphism markers. Percentages on the axis represent the variance explained by each coordinate. **(B)** Maximum likelihood reconstruction of 423 accessions of Peruvian maize germplasm using 14,235 single nucleotide polymorphism markers. Round symbol on nodes represents bootstrap support, with only values higher than 90% shown. Accessions were labeled according to their Peruvian geographic department of origin.

Supplementary Figure 8
**(A)** Principal coordinate analysis of 423 accessions of Peruvian maize germplasm using 14,235 single nucleotide polymorphism markers. Percentages on the axis represent the variance explained by each coordinate. **(B)** Maximum likelihood reconstruction of 423 accessions of Peruvian maize germplasm using 14,235 single nucleotide polymorphism markers. Round symbol on nodes represents bootstrap support, with only values higher than 90% shown. Accessions were labeled according to their geographic zone of origin.

Supplementary Figure 9
**(A)** Population structure of 423 maize accessions based on 14,235 single nucleotide polymorphism markers. Each accession is represented by a vertical bar, and each color corresponds to a population (two in total). **(B)** Population structure of 423 maize accessions based on 14,235 single nucleotide polymorphism markers. Each accession is represented by a vertical bar, and each color corresponds to a population (four in total).

Supplementary Figure 10 Cluster assignation based on STRUCTURE results for Peruvian maize accessions according to their geographic origin.
