# Supplementary figures and images for: Genotyping by sequencing reveals the genetic diversity and population structure of Peruvian highland maize races
Source: Front Plant Sci. 2025 Feb 25;16:1526670. doi: 10.3389/fpls.2025.1526670 (PMC11893605; doi:10.3389/fpls.2025.1526670)

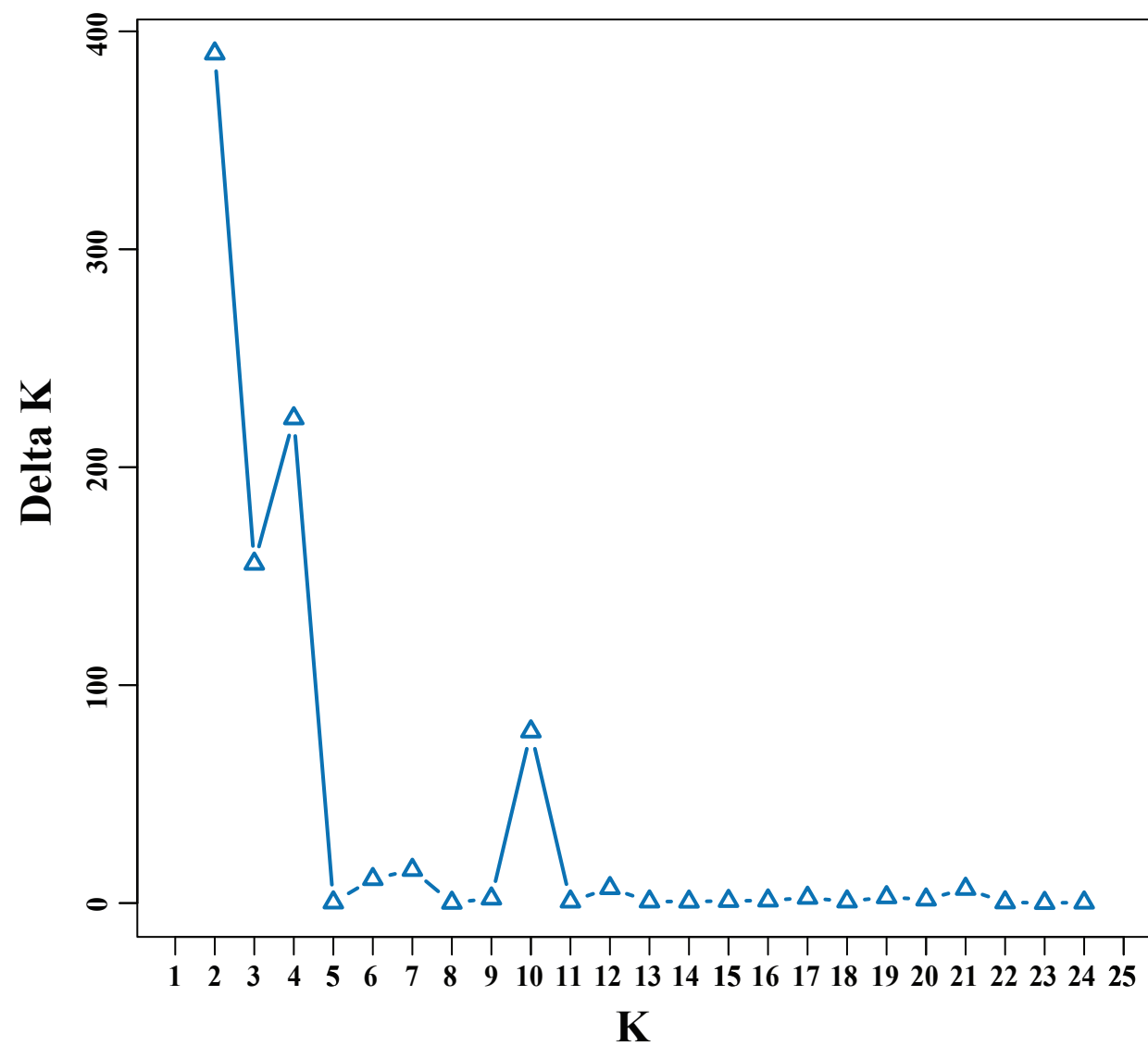

Supplement: Supplementary Figure 1 — Plot of Delta K (ΔK). Twenty-five populations were considered in a data set of 14,235 SNPs and 423 accessions of Peruvian maize germplasm. [file DataSheet1.pdf]

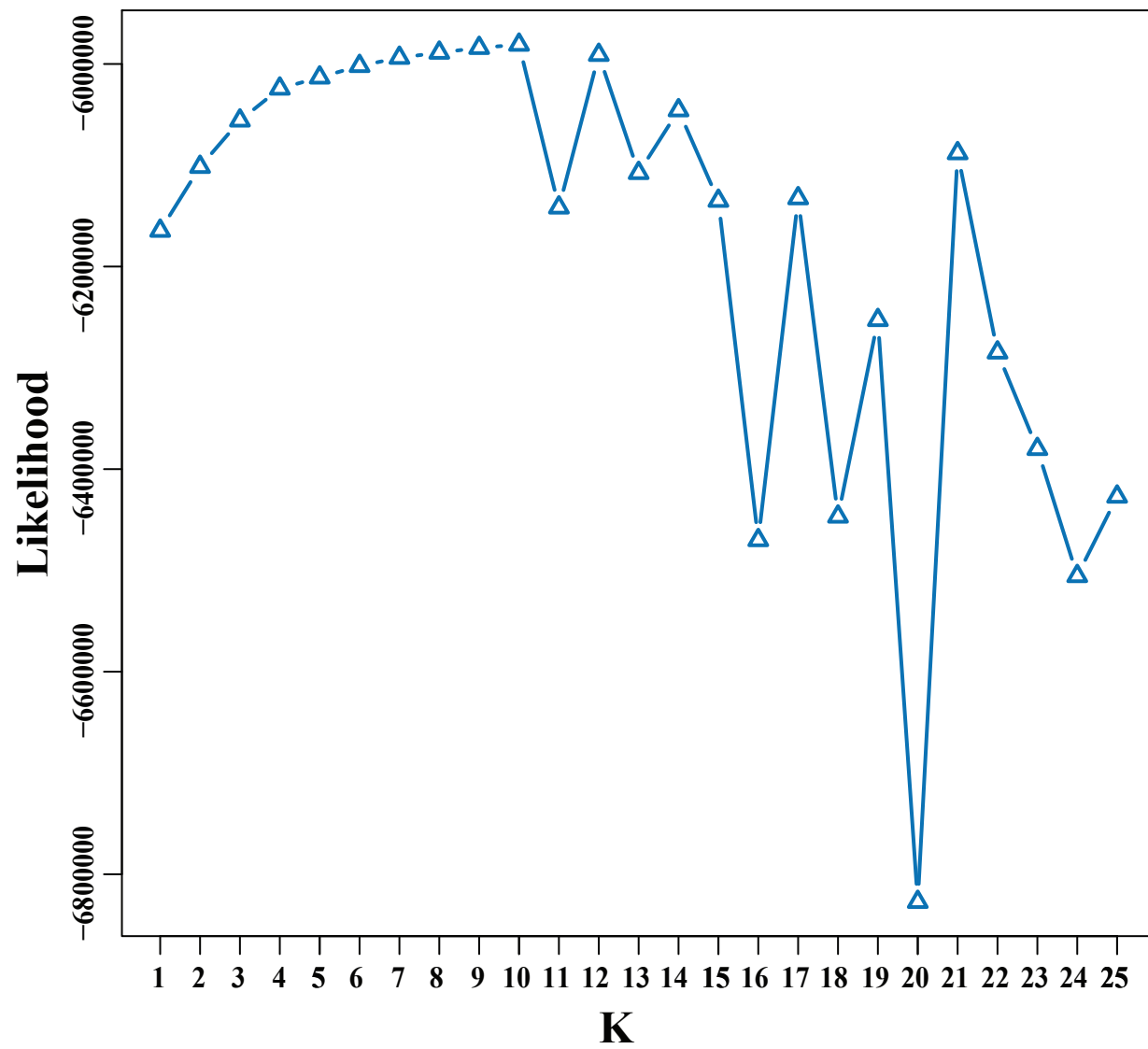

Supplement: Supplementary Figure 2 — Plot of the log likelihood for K ranging from 1 to 25. [file DataSheet2.pdf]

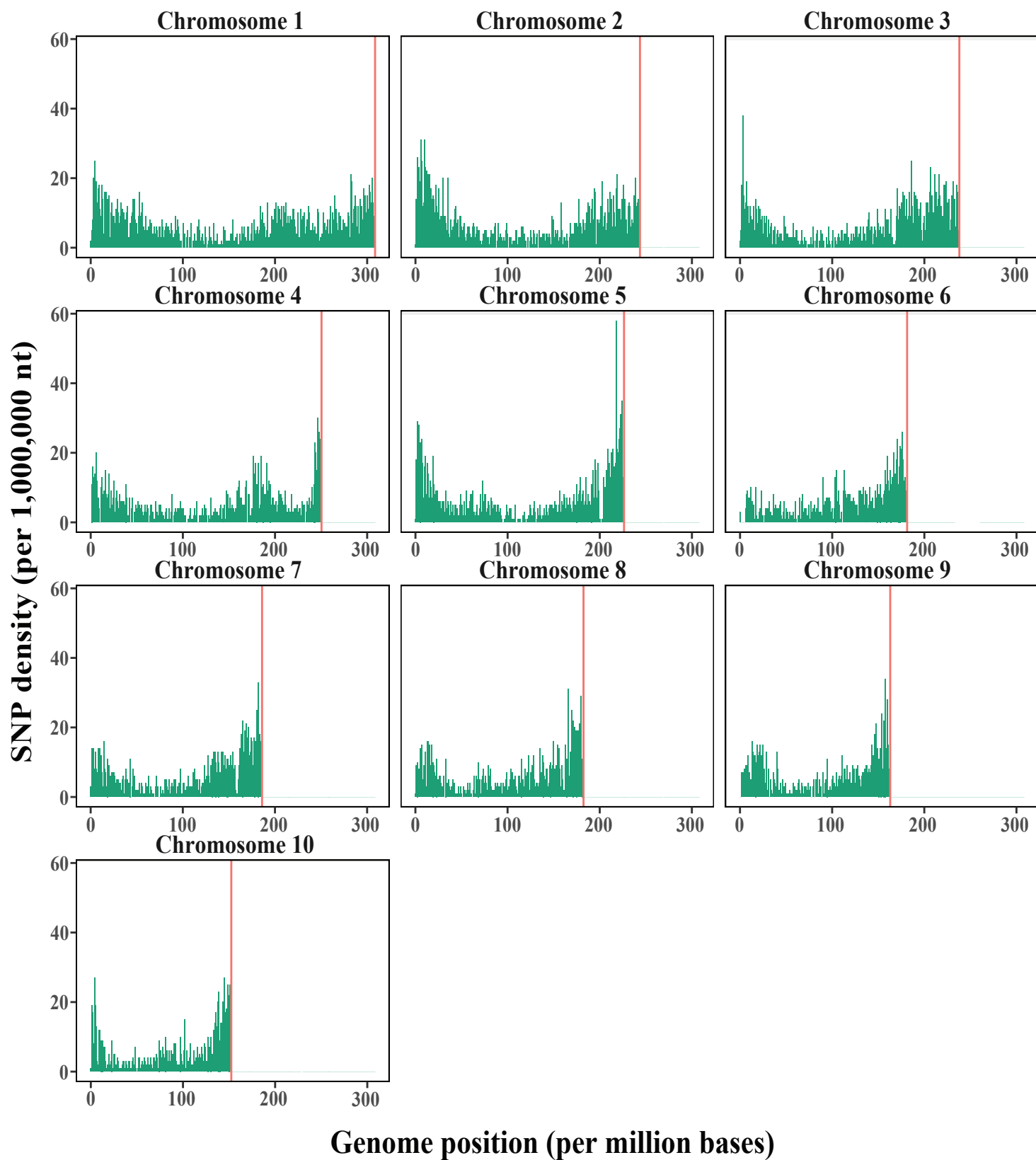

Supplement: Supplementary Figure 3 — Density and distribution of 14,235 single nucleotide polymorphism markers on the 10 maize chromosomes. Red bars denote the end of chromosome; nt refers to nucleotide. [file DataSheet3.pdf]

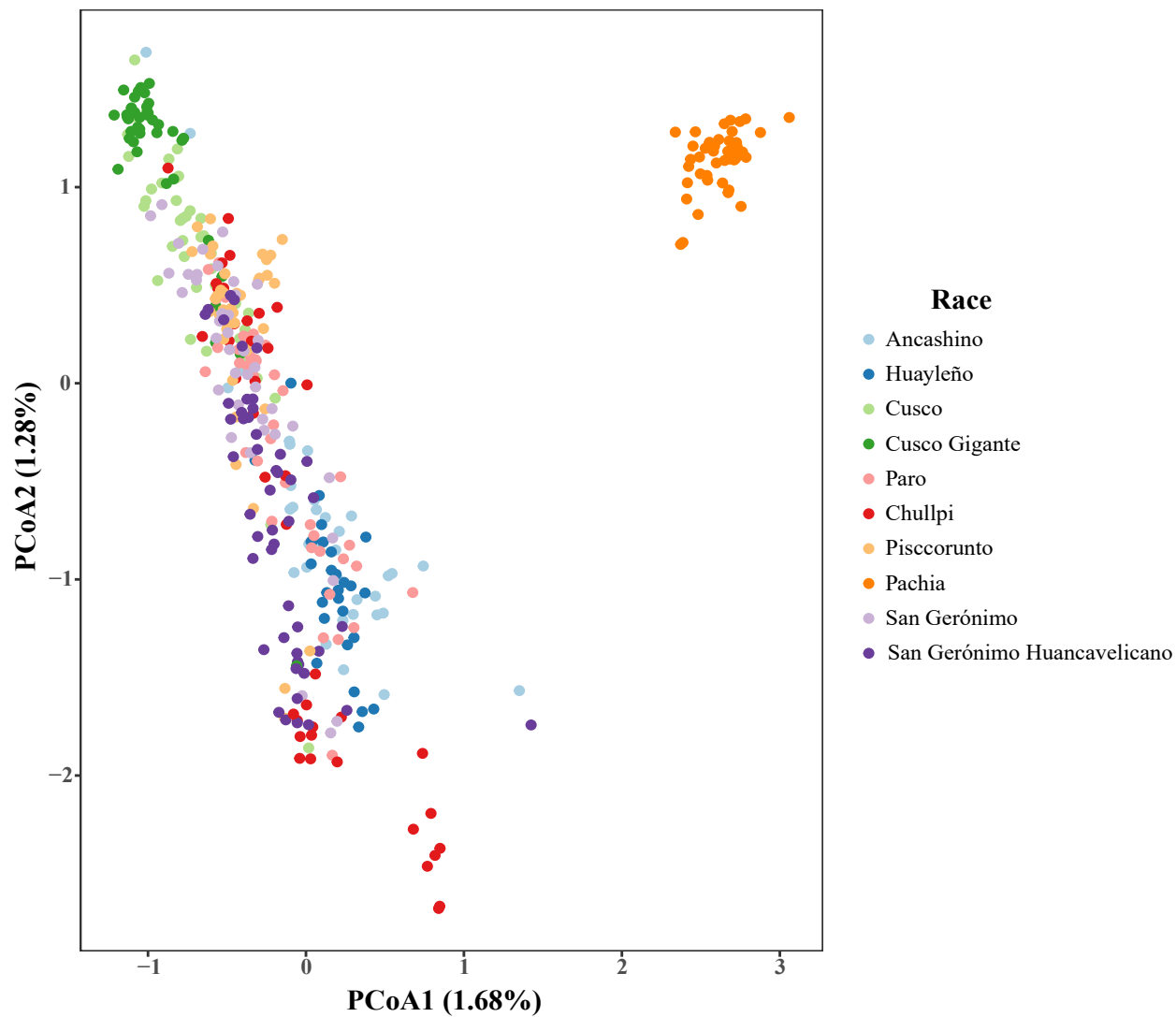

Supplement: Supplementary Figure 4 — Principal coordinate analysis of 406 accessions of Peruvian maize germplasm (improved maize not included) using 14,235 single nucleotide polymorphism markers. Percentages on the axis represent the variance explained by each coordinate. [file DataSheet4.pdf]

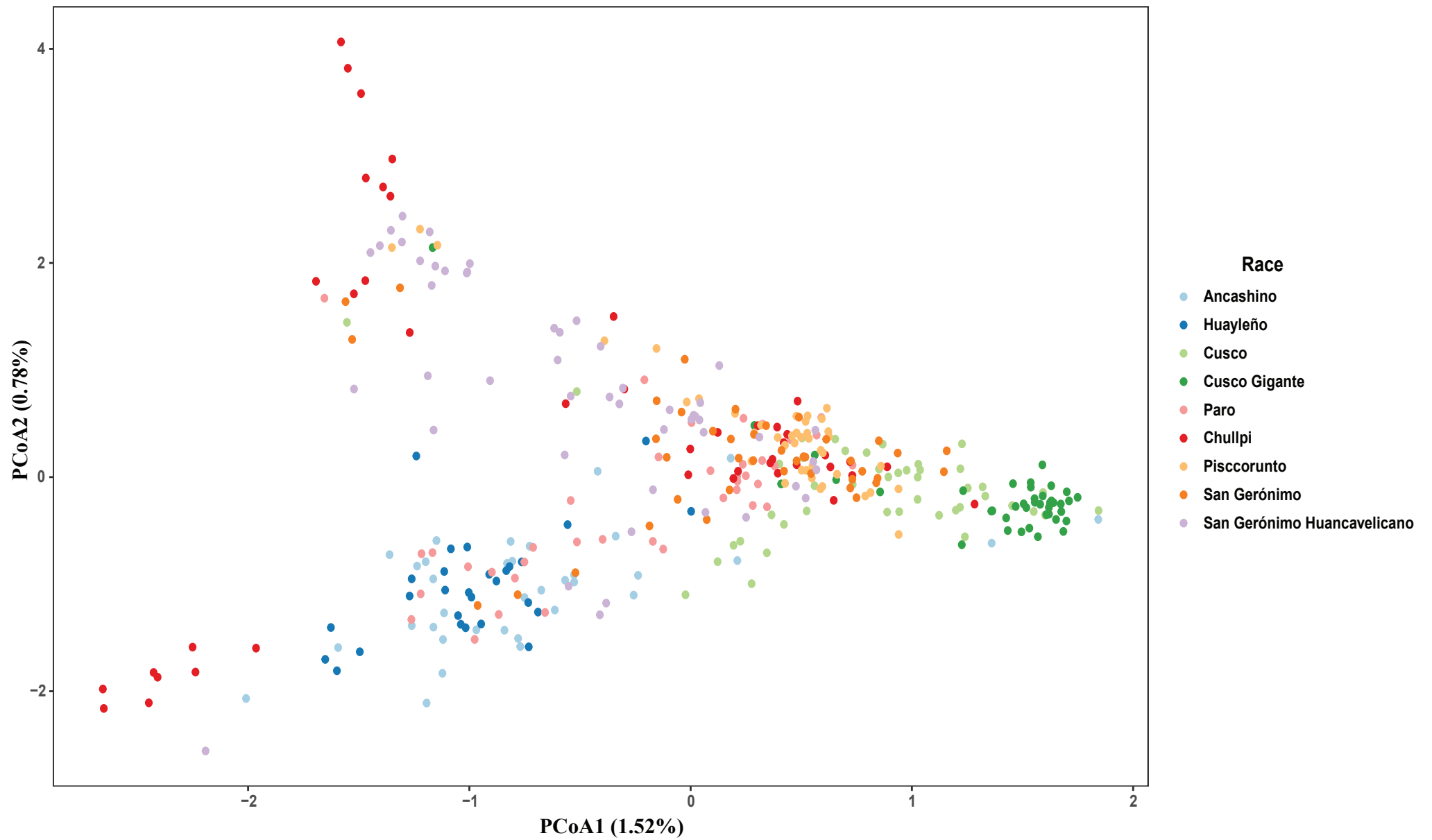

Supplement: Supplementary Figure 5 — Principal coordinate analysis of 363 accessions of Peruvian maize germplasm (improved maize and sub-race Pachia not included) using 14,235 single nucleotide polymorphism markers. Percentages on the axis represent the variance explained by each coordinate. [file DataSheet5.pdf]

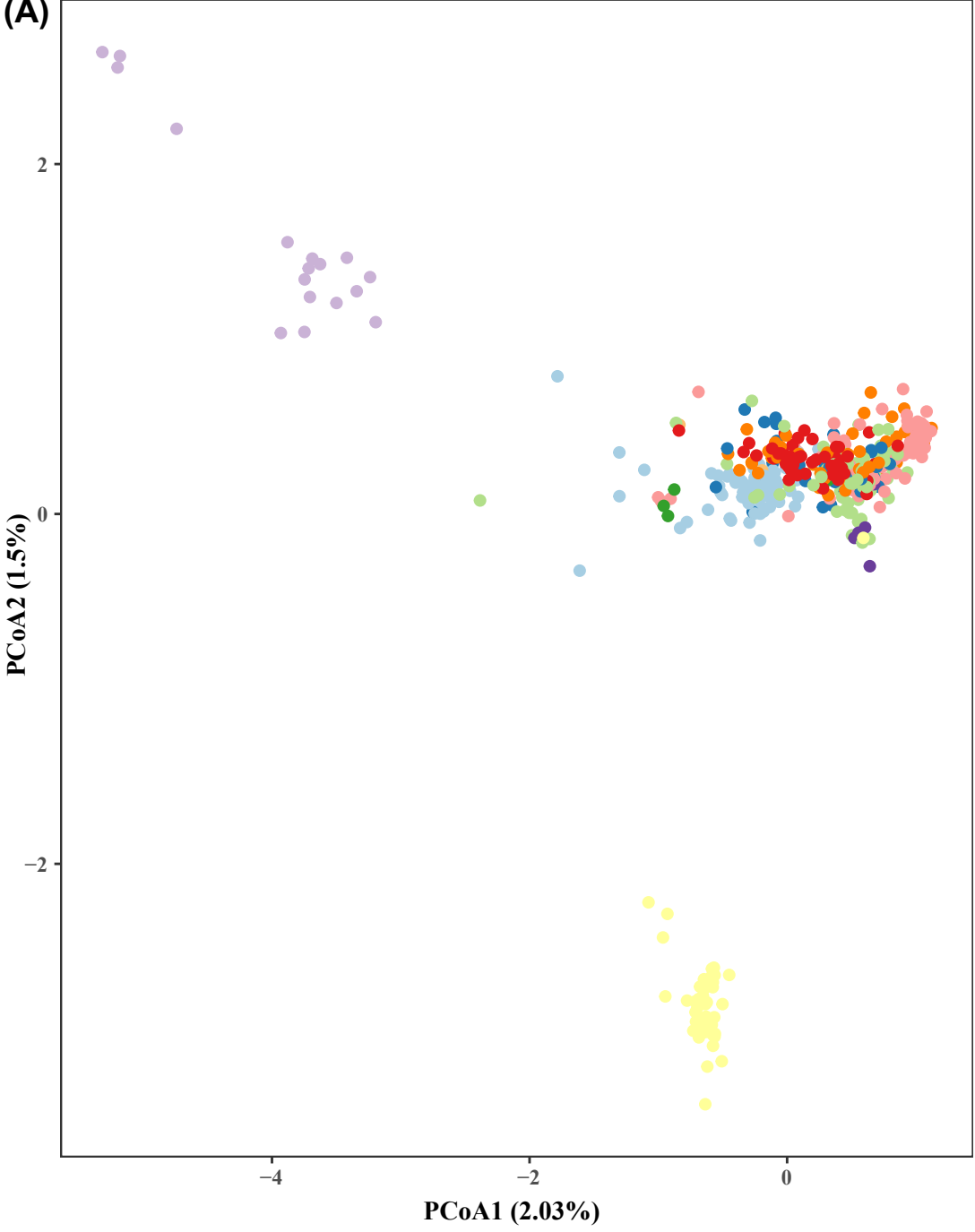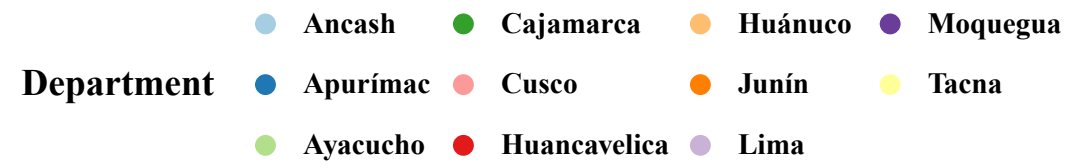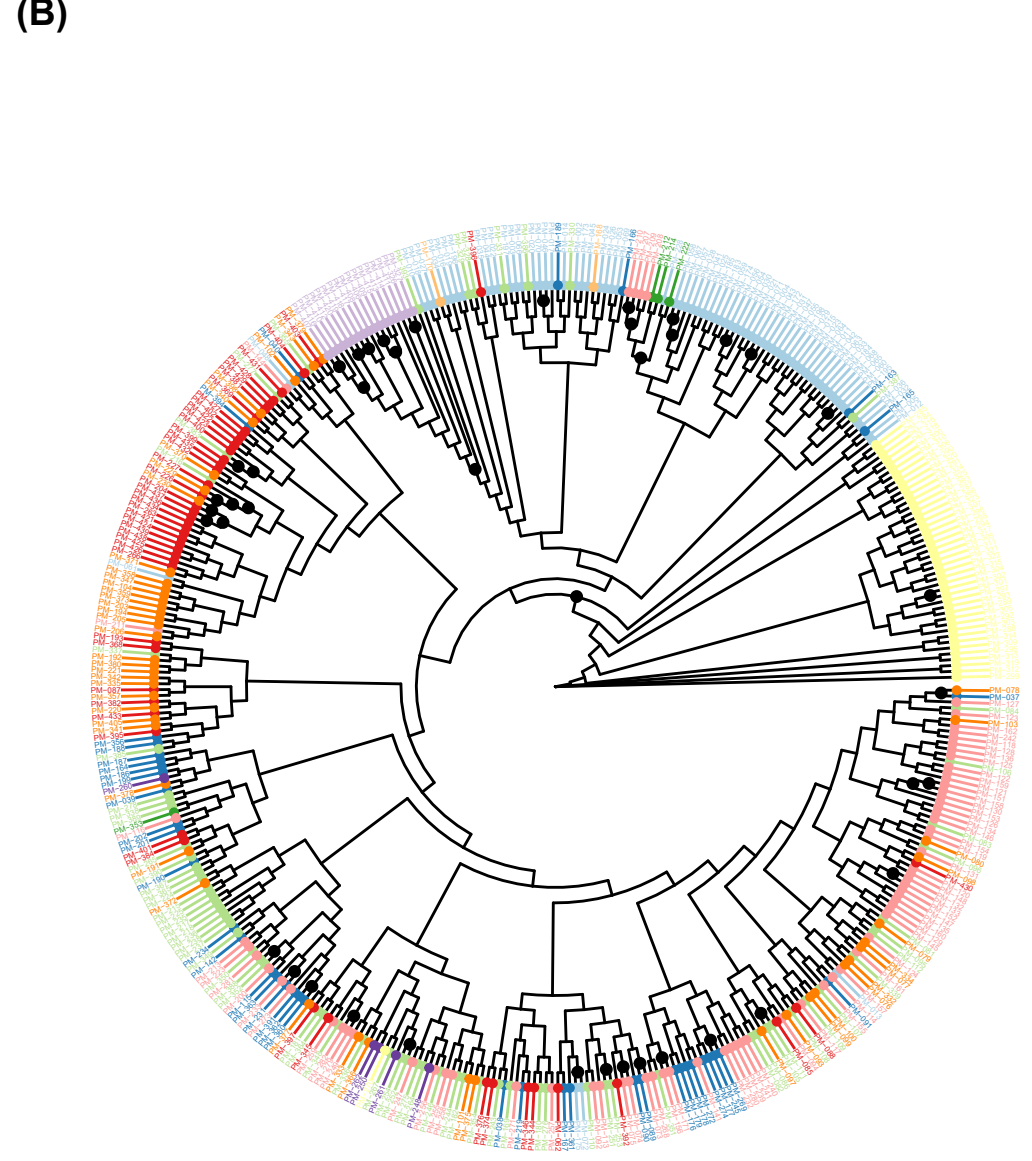

Supplement: Supplementary Figure 7 — (A) Principal coordinate analysis of 423 accessions of Peruvian maize germplasm using 14,235 single nucleotide polymorphism markers. Percentages on the axis represent the variance explained by each coordinate. (B) Maximum likelihood reconstruction of 423 accessions of Peruvian maize germplasm using 14,235 single nucleotide polymorphism markers. Round symbol on nodes represents bootstrap support, with only values higher than 90% shown. Accessions were labeled according to their Peruvian geographic department of origin. [file DataSheet7.pdf]

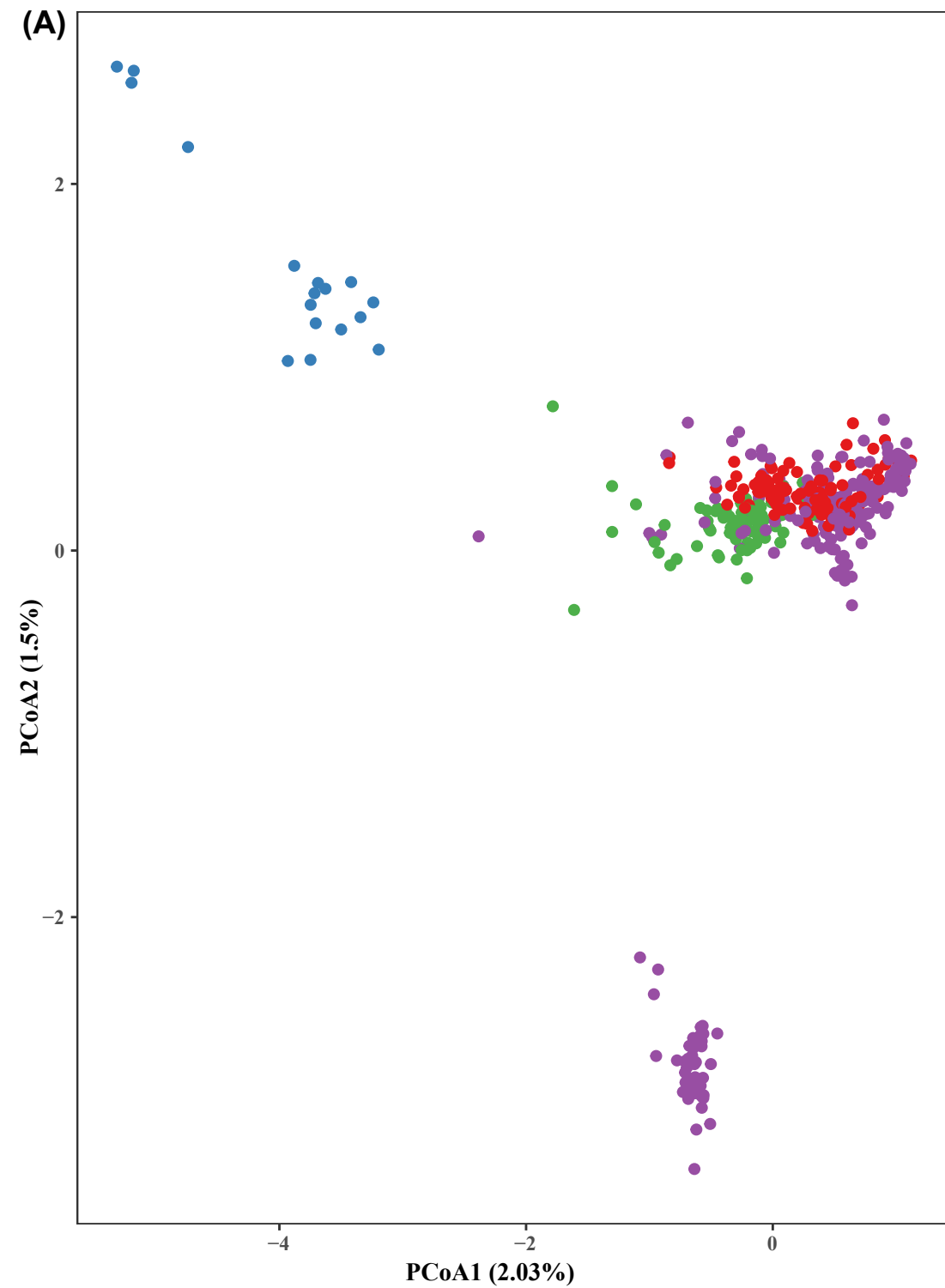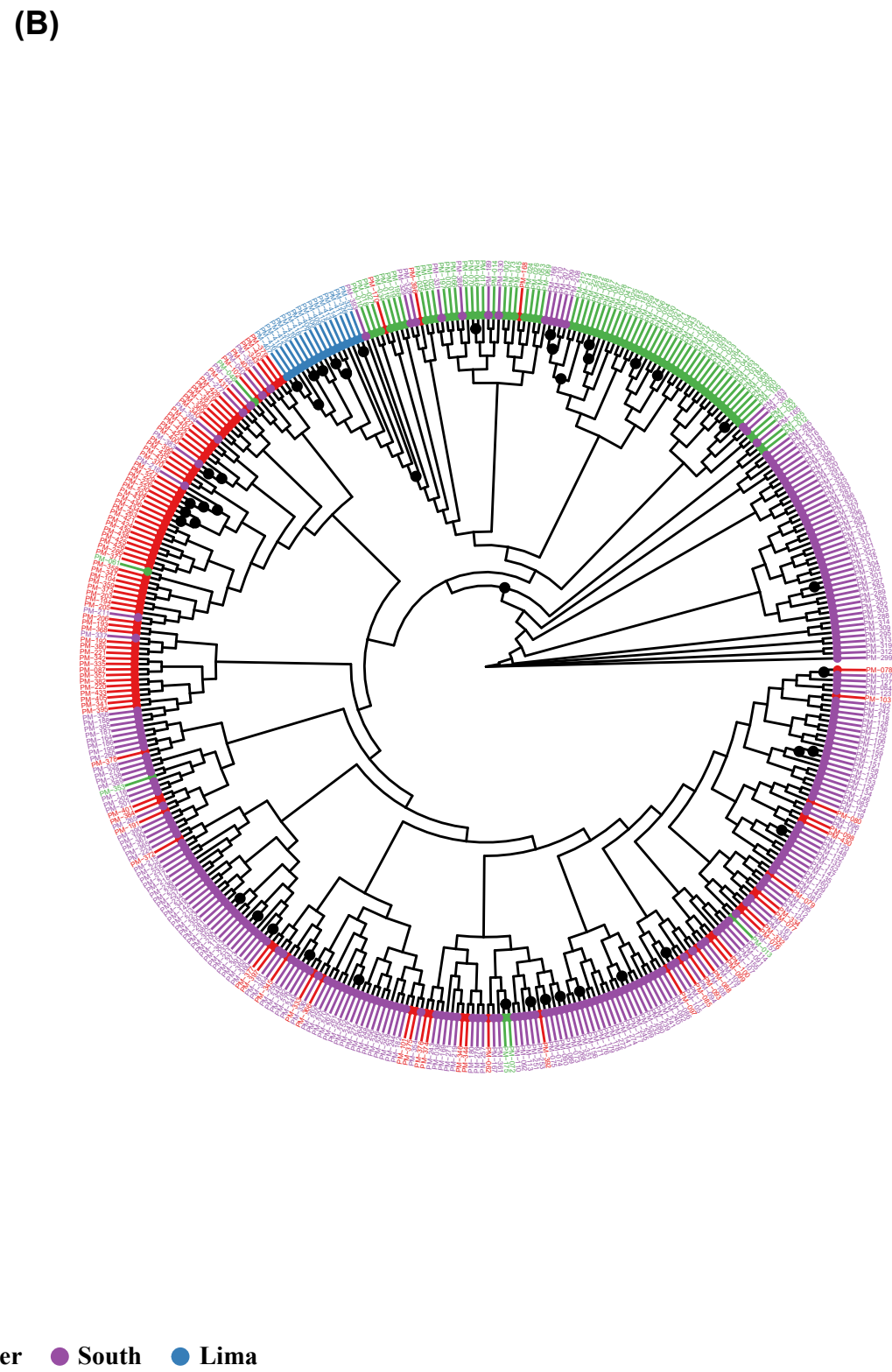

Supplement: Supplementary Figure 8 — (A) Principal coordinate analysis of 423 accessions of Peruvian maize germplasm using 14,235 single nucleotide polymorphism markers. Percentages on the axis represent the variance explained by each coordinate. (B) Maximum likelihood reconstruction of 423 accessions of Peruvian maize germplasm using 14,235 single nucleotide polymorphism markers. Round symbol on nodes represents bootstrap support, with only values higher than 90% shown. Accessions were labeled according to their geographic zone of origin. [file DataSheet8.pdf]

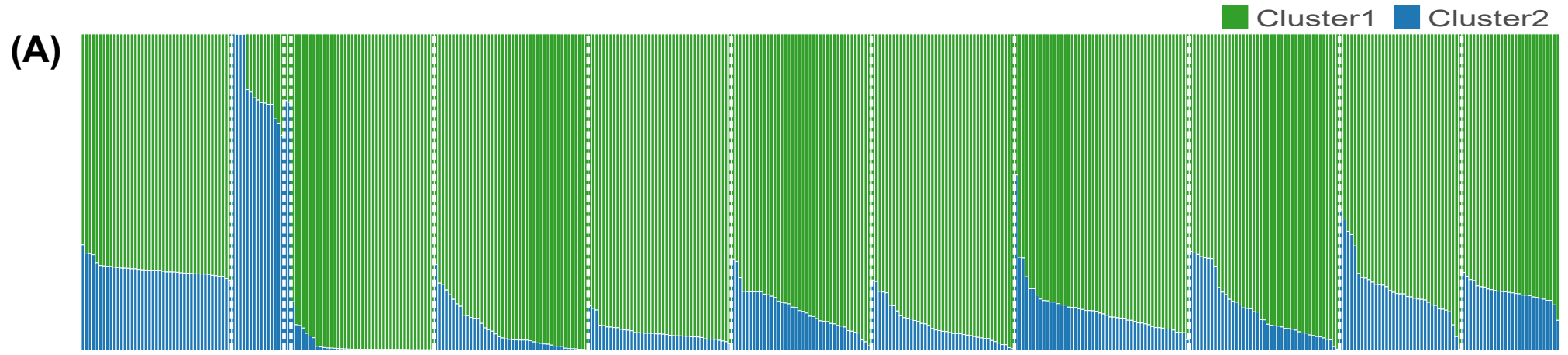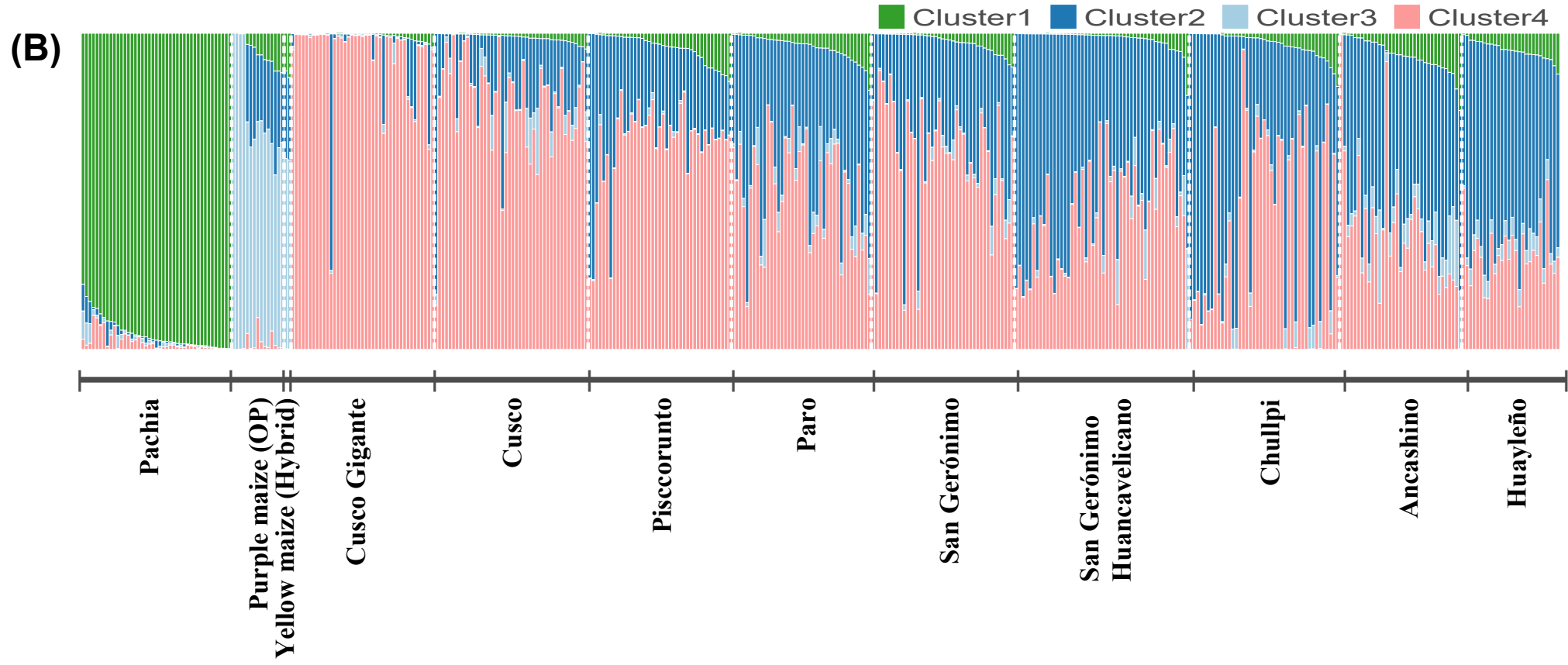

Supplement: Supplementary Figure 9 — (A) Population structure of 423 maize accessions based on 14,235 single nucleotide polymorphism markers. Each accession is represented by a vertical bar, and each color corresponds to a population (two in total). (B) Population structure of 423 maize accessions based on 14,235 single nucleotide polymorphism markers. Each accession is represented by a vertical bar, and each color corresponds to a population (four in total). [file DataSheet9.pdf]

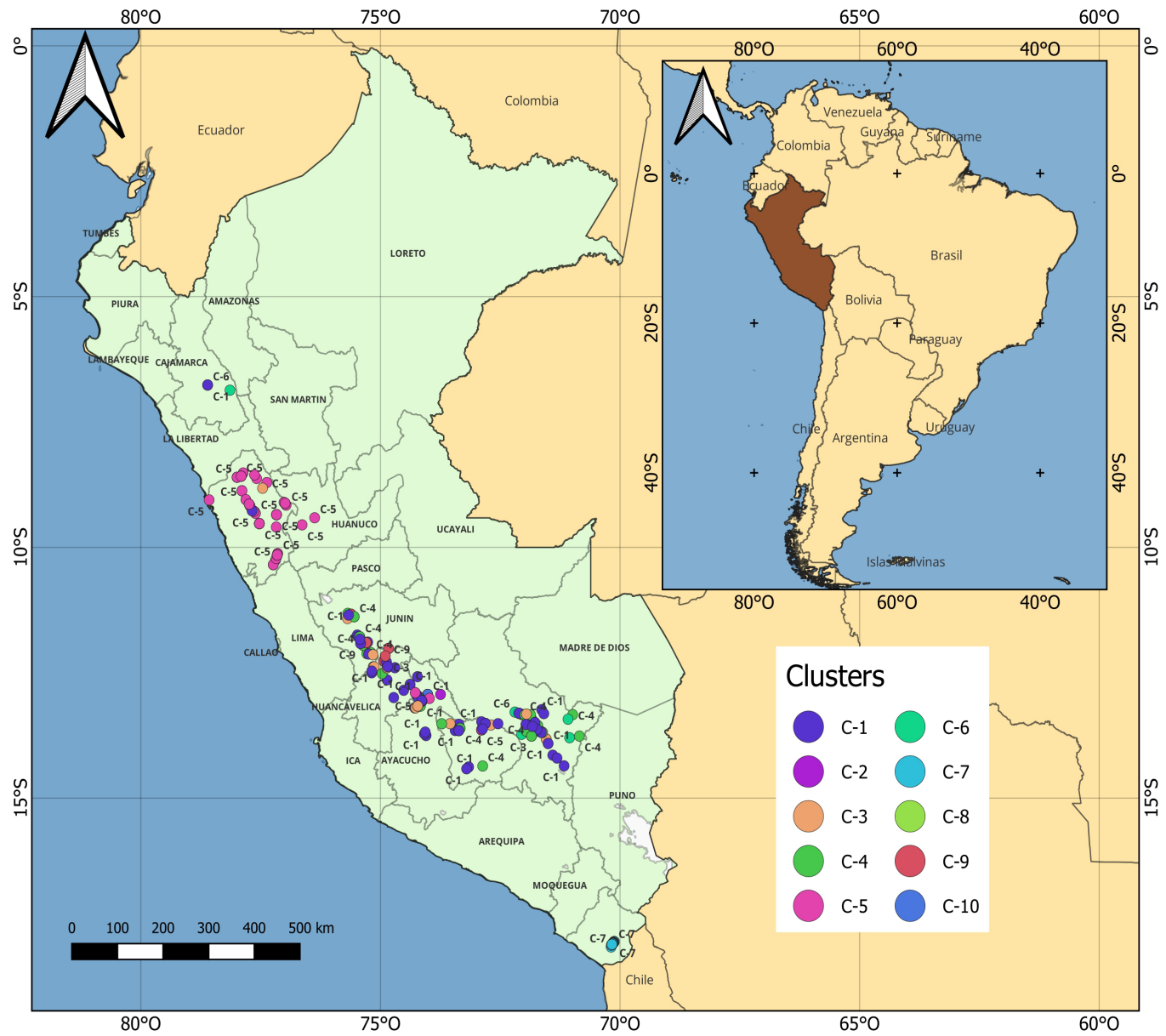

Supplement: Supplementary Figure 10 — Cluster assignation based on STRUCTURE results for Peruvian maize accessions according to their geographic origin. [file DataSheet10.pdf]
