# Supplementary material for: Genotyping by sequencing reveals the genetic diversity and population structure of Peruvian highland maize races
Source: Front Plant Sci. 2025 Feb 25;16:1526670. doi: 10.3389/fpls.2025.1526670 (PMC11893605; doi:10.3389/fpls.2025.1526670)
Supplement: Supplementary Figure 6 — Dendrogram based on Provesti’s genetic distance and the neighbor-joining clustering method of 423 accessions of Peruvian maize germplasm using 14,235 single nucleotide polymorphisms markers. Numbers above the branches represent bootstrap values, with only values higher than 90% shown. [file DataSheet6.pdf]

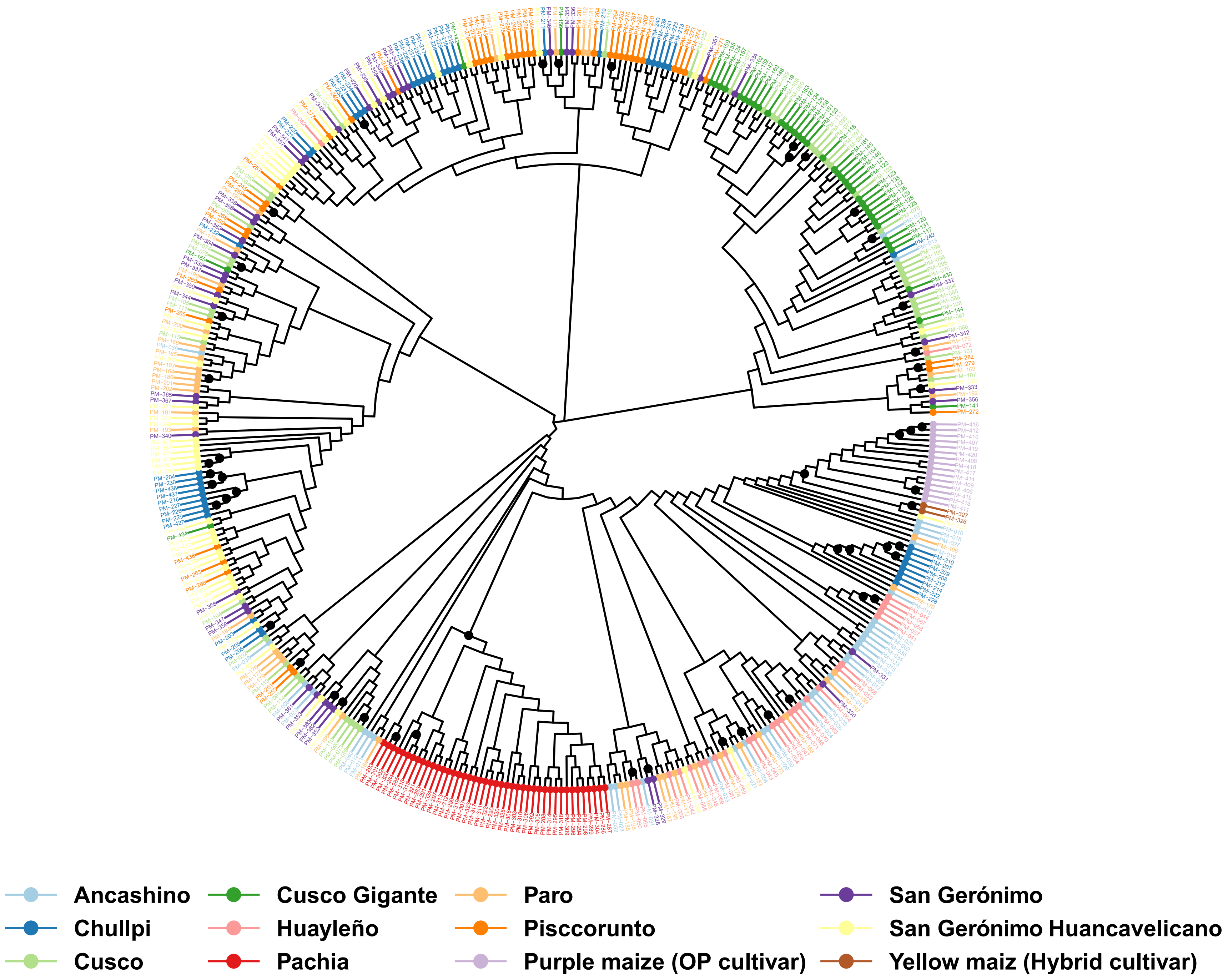
Ancashino
Chullpi
Cusco
Cusco Gigante
Huayleño
Pachia
Paro
Pisccorunto
Purple maize (OP cultivar)
San Gerónimo
San Gerónimo Huancavelicano
Yellow maiz (Hybrid cultivar)
